# When Love Comes at a Cost: Mental Health Outcomes in Older Adults Providing Grandparental Care

**DOI:** 10.3390/healthcare13141685

**Published:** 2025-07-13

**Authors:** Han Hu, Wei Zeng, Ran Liu

**Affiliations:** School of Public Policy and Administration, Xi’an Jiaotong University, Xi’an 710049, China; huhan112@xjtu.edu.cn (H.H.); 2224212562@stu.xjtu.edu.cn (W.Z.)

**Keywords:** grandparental care, mental health of the elderly, intergenerational exchange theory, intergenerational support

## Abstract

Background/Objectives: Against the backdrop of increasing global aging and the trans-formation of family structures, grandparental caregiving has become commonplace, and its impact on the mental health of older persons is of great concern. Methods: Based on data from the 2023 Xi’an Jiaotong University Urban and Rural Elderly Family Support and Psychological Condition Survey, this study analyzed the impact of grandparental care-giving behaviors on the mental health of the elderly through the Psychological Condition Measurement Scale (PCMS), and comprehensively assessed the presence, intensity, and heterogeneous impact of grandparental caregiving behaviors by gender by applying linear regression modeling, the Propensity Score Matching (PSM) method, and the Instrumental Variables Method (IVM). Results: Grandparental care has a significant positive effect on the mental health of older people, but this positive effect diminishes as the intensity of care increases. The results of the sub-sample estimation show that grandparental caregiving has a positive effect on men’s and low-intensity caregiving on women’s mental health, but high-intensity caregiving has a negative effect on women’s mental health. In addition, in-tergenerational financial support and intergenerational emotional comfort play an im-portant mediating role between grandparental caregiving and the mental health of older persons, in line with the explanatory framework of intergenerational exchange theory. Conclusions: It is recommended that the Government support grandparental care for the elderly at the financial, social security, and policy levels; that society build a diversified system of elderly care services and strengthen public childcare services; and that families establish a value identity of two-way support.

## 1. Introduction

Aging has become a major issue for the world’s livelihood. According to the World Health Organization, by 2050, the global population aged 60 and above is expected to account for more than 22% of the total population [1]. By the end of 2024, China’s population aged 60 and above will be 310.31 million, already accounting for 22.0% of the country’s population, making the elderly an important part of China’s population [2]. The wave of population ageing is not only reshaping the age structure of society, but also profoundly rewriting the mode of operation of family functions. With the aging of the population and the nuclearization of the family structure, the contradiction between childcare and work for young parents has become increasingly prominent. In the traditional Chinese family structure, it is common to see three or even four generations of grandchildren living in the same household, and the Confucian culture of filial piety emphasizes the responsibility of elders to care for and teach their juniors, making this close-knit family model a natural breeding ground for grandparents to provide care. With their rich experience and time advantage, grandparent groups have gradually changed from “cared-for” to “caregivers”, and grandparental care has become an important form of family function reconstruction in an aging society. Guzma found, based on data from two large national surveys, that 47% of American grandparents are involved in childcare [3]. The 2017 International Longevity Center report noted that two-thirds of grandparents in the United Kingdom make economic contributions to the growth of their grandchildren [4]. In China, statistics show that 51.7% of seniors provide grandparental care [5]. With the implementation of China’s comprehensive “three-child” policy, the miniaturization of the family structure, and the mainstreaming of traditional family pensions, caring for grandchildren by the elderly has become a realistic choice for many families. The State Council of the People’s Republic of China’s Guiding Opinions on Further Optimizing and Improving Positive Childbirth Support Policies and the National Health Plan for the Fourteenth Five-Year Plan both explicitly state that “localities are encouraged to take active measures to support grandparental care, family mutual assistance and other modes of care”.

Mental health is an important part of aging. Data from the World Health Organization 2023 show that globally, about 14% of older people aged 60 and above suffer from depressive moods [6]. The Blue Book “China Aging Development Report 2024—Mental Health Status of Chinese Elderly People” shows that 26.4% of older people in China suffer from varying degrees of depressive symptoms, of whom 6.2% have moderately severe depressive symptoms [7]. The 2019 State Council of the People’s Republic of China’s “Guiding Opinions on Promoting the Action for a Healthy China” explicitly points out that it is necessary to actively carry out work related to mental health and geriatric health promotion with a view to healthy aging [8]. Consequently, paying attention to the mental health of the elderly and achieving a sense of well-being in old age have become important tasks in the comprehensive promotion of healthy ageing.

The way in which the elderly contribute to grandparental care by playing the role of “residual heat” not only brings them a sense of freshness and enhances intergenerational relationships, but also triggers the risk of being forced to adapt to the care environment and change their lifestyle. Theoretically, grandparental caregiving reduces the pressure on parents, improves the well-being of the whole family, and improves one’s psychological well-being. However, the fact that grandfathers and grandmothers have different roles and positions in the family can lead to significant heterogeneity in grandparental caregiving behaviors among older adults. Therefore, it is important to explore the impact of grandparental caregiving and the resulting changes in intergenerational relationships on the psychological health of the elderly, to examine the heterogeneity of the group caused by gender difference [8], to discuss whether grandparental caregiving can achieve a mutually beneficial situation that not only promotes the birth of a family, but also protects the rights and interests of the elderly, and to put forward targeted recommendations based on the strategy of positive aging.

On the eve of the Spring Festival in 2023, General Secretary Xi Jinping mentioned in his speech during his condolences to the cadres and masses at the grassroots level that “our society is aging more and more, and we must let the elderly have a happy old age.” This paper analyzes and compares the mental health status of three categories of older adults, namely, no grandparental care, low-intensity care, and high-intensity care, by using the Psychological Condition Rating Scale (PCRS), examines the extent to which grandparental caregiving affects older adults’ mental health, analyzes the mechanisms of this impact, and provides a comprehensive assessment taking into account endogeneity and self-selective bias, to provide a more nuanced explanation of the theoretical perspectives of grandparental caregiving for older adults.

## 2. Literature Review and Theoretical Framework

### 2.1. The Internalization of Responsibility Theory and the Cultural Heterogeneity of Grandparental Caregiving

In countries such as the United States, the United Kingdom, and Germany, where the ideology of individualization has been emphasized, the process of individualization and patterns of economic practice have often weakened the social value and identity of grandparents [4] (p. 2). In grandparental care models, grandparents are more often given the role of “being there” and “not interfering”, i.e., being available while not disrupting the parent–child relationship [9]. As a result, grandparents are often in a relatively passive cultural norm. In contrast, in countries with strong generational complexes such as Italy, Greece, and especially China, filial piety and the duty to care for parents have redefined the status of grandparents in the family [10]. Among other things, the family-oriented culture emphasizes the bond of blood kinship and the patriarchal principle as dominant, forming the status regulations and behavioral norms of superiority, inferiority, superiority and superiority, and the order of seniority and juniority [11]. This has given rise to the internalized theory of responsibility, which states that parents and grandparents have an unconditional duty of care to each other and that the provision of grandparental care is also an internalized family responsibility [12], while refusal to care may result in negative comments from neighbors or relatives. This cultural difference in the responsibilities of grandparental caregiving actors can backfire on the actors themselves. Chan et al. [13] note that grandparental caregiving intensity has a concave-curve relationship with health and well-being, with optimal intensity varying by culture: complementary caregiving is beneficial to dual-earner families in places such as Europe and the United States; economic resources in East Asia buffer against the adverse effects of primary caregiving on well-being; and in the United States, there are differences in outcomes by ethnicity, with the relationship compounded by family roles and cultural differences.

### 2.2. The Impact of Grandparental Care on the Mental Health of Older Persons

Research on the impact of grandparental caregiving on the mental health of older adults is divided, and several competing theories currently exist in the academic community.

#### 2.2.1. Role Optimization Theory

Role optimization theory suggests that the role of grandparental caregiving, as an important component of the multiple social roles of older adults, contributes to positive emotional experiences, mitigates pressures and risks from other social roles, and enhances their overall psychological well-being [14]. Based on the “use it or lose it” principle, taking on active caregiving roles and remaining socially engaged can slow the rate of cognitive decline and reduce the risk of cognitive impairment or depression in older persons [15,16]. This provides a theoretical basis for the positive relationship between grandparental caregiving and older adults’ mental health: Jianguo Zhao [8] (p. 2) demonstrated that grandparental caregiving significantly promotes older adults’ mental health based on Maslow’s hierarchy of needs theory; Shen et al. [17] showed that older adults who provide grandparental care have better psychological well-being; Shuzhuo Li et al. [18] showed that older adults’ financial support for their grandchildren is beneficial to their subjective health; and Fengzhen Tsai [19] noted that elders who continued or started caring for their grandchildren were happier and enjoyed life more than those who did not.

#### 2.2.2. Role Burden Theory

Role Burden Theory suggests that when older adults have difficulty in balancing role conflicts under the demands of multiple roles, or that when their role load exceeds their carrying capacity, they are prone to negative emotional experiences, and that this long-standing “chronic stressor” can cause progressive damage to their health [20]. The elderly who provide grandparental care have a new role as “grandparents” and are faced with the task of caring for their grandchildren, there will be a certain amount of conflict, transformation, and enhancement between the multiple roles, and the interactive process will also have an impact on their mental health that cannot be ignored [21]. This theory supports the negative correlation between grandparental care and the mental health of the elderly: Lindsey et al. [22] point out that grandparents who are involved in multiple family roles have more significant symptoms of depression, which may be related to the conflict and stress between their different roles; Hughes et al. [23] and Musil et al. [24] point out that grandparental caregiving is likely to lead to the depletion of the energy of middle-aged and elderly people and increase the burden of family labor, which may lead to a significant decline in their mental health, which may in turn increase the risk of depression. Li et al. [25] and Whitley et al. [26] point out that grandparental caregiving reduces the frequency of daily social interaction among older adults, leading to economic depletion and family tension, which may cause physical or psychological stress to older adults.

#### 2.2.3. The Theory of Proportionate Labor

The theory of proportionate labor points out that when the individual labor input is in the reasonable range, the pressure brought by labor is in the tolerable range, and can be transformed into positive motivation to optimize behavioral performance; when the labor input is insufficient or excessive, this will trigger physical and mental stress and accumulate over time, and once it exceeds the threshold up to which the individual can withstand it, it will damage their health, i.e., the correlation between the labor input and the pressure is an inverted “U” [27]. The economics of diminishing marginal utility can also explain this theory: the mental health effects of grandparental care show dynamic changes with the intensity and duration of care and individual resources, and over-investment can be transformed into a burden due to resource depletion. This provides theoretical support for the complex relationship between grandparental care and the mental health of the elderly: Baoqing Han [28] and Yuyang Li et al. [29] point out that as the duration of grandparental care grows, the positive effects of grandparental care on the mental health of the elderly decline, showing an inverted U-shaped trend of change. Qinghong He [30] believes that grandparental care significantly enhances the well-being of grandchildren under the age of 60, and promotes attenuation or even inhibition at the age of 60 and above. Lianjie Wang [31] showed that excessive caregiving is detrimental to health and is a complex experience that is both tiring and enjoyable. Runnan Zhi [32] argues that older people’s mental health is significantly enhanced only when they are within their means and subjectively willing to provide financial support to their children.

#### 2.2.4. The Grandmother Hypothesis—An Analysis of Gender Heterogeneity

In the last decade, based on an interdisciplinary perspective that combines social science and evolutionary theory, Hawkes et al. [33] have proposed the “grandmother hypothesis”, which suggests that the evolutionary roots of female menopause may lie in the motivation of older women to increase their survival rates by helping to raise their grandchildren. Lahdenperä et al. [34] further suggest that the role of the grandmother has become a defining characteristic of the human family, regardless of the evolutionary reasons for menopause. Although the theory is still controversial, it provides interdisciplinary insights into understanding the more important role played by female older adults in grandparental caregiving. Grandparental caregiving also varies significantly from study to study in terms of the extent of its impact on gender. Lianjie Wang [31] (p. 4) notes that grandparental caregiving has a more significant impact on female older adults. Yue Hong et al. [35], after considering subgroup differences, noted that the mental health benefits of continuing care were particularly pronounced among urban grandmothers. He Guangye et al. [36] concluded that although women are more involved in intergenerational parenting than men, intergenerational parenting does not have a significant effect on women’s self-assessed health and reduces men’s overall self-assessed health. From the perspective of rural elderly migration, Jin Xiaoyi et al. [37] concluded that the grandparental caregiving behaviors of male floating elderly was lower than that of female floating elderly in terms of life satisfaction.

### 2.3. Intergenerational Exchange Theory—An Exploration of the Mechanisms of Influence

Unlike the Western “relay model”, the intergenerational exchange relationship in China is based on the principle of “raising children for old age”, which is balanced and reciprocal and generally shows the characteristics of “two-way” and “feeding back” [38,39,40]. Guangzong Mu et al. [41] clearly define that the support of the younger generation includes both economic support and spiritual support. Based on this, the theory of intergenerational exchange suggests that in an environment where formal social support is insufficient, family members tend to form a mutual support network to maximize the satisfaction of individual and family needs through the exchange of resources [42]. Currently, family old-age care is still the main mode in China, and since the elderly have declining labor ability and lower income levels, by undertaking grandparental care and other activities, they can reduce the time and energy spent on caring for their children by their offspring, as well as lowering the opportunity cost of their offspring, thus realizing the optimal allocation of resources within the family. In exchange, the elderly can obtain material support and spiritual comfort from their children [43]. This type of family cooperation system is based on the reciprocity mechanism, forming an intergenerational resource exchange model with implicit contractual attributes [44]. In other words, older people often make intergenerational payments to their children by taking on the care of their grandchildren in return for the support provided by their children in their old age [12] (p. 3). Relevant studies have confirmed that older adults who care for their grandchildren are more likely to receive intergenerational feedback from their children, including financial support and spiritual comfort [42,45] (p. 5).

### 2.4. Research Problem

Based on a multidimensional theoretical perspective, existing studies have systematically explored grandparental caregiving from three perspectives: cultural differences, specific influences, and influence mechanisms. From a comparative cultural perspective, the internalization of the responsibility theory reveals the essential differences between Chinese and Western grandparental caregiving, with the West emphasizing the complementary and passive roles of grandparents, while China, influenced by its family-oriented culture, internalizes grandparental caregiving as a family responsibility. This difference confirms that exploring grandparental caregiving in the Chinese context has some specificity and theoretical significance in the world. In terms of specific impacts, the competing explanatory frameworks of optimization, burden, and moderate labor theories, as well as the gender heterogeneity explained by the grandmother hypothesis, highlight the complexity of the grandparental caregiving effect. This complexity is not yet recognized in the academic community, and more comprehensive sub-sample estimates are lacking. In terms of the impact mechanism, the intergenerational exchange theory provides a possible explanatory framework, but its appropriateness has not been fully verified. Thus, this paper, based on the previous work and on the cultural background of China’s state-orientation, attempts to explore the following questions:

First, what kind of relationship does grandparental caregiving present with older adults’ mental health?

Second, does grandparental caregiving have a different impact on mental health for older persons of different genders?

Third, what factors play a mechanistic role in the impact of grandparental caregiving on the mental health of older persons, and what specific roles do they play?

### 2.5. Theoretical Framework

In order to answer the three questions raised in the previous section, firstly, this paper starts by discussing the presence and intensity of grandparental caregiving behaviors among older adults, examining whether there are differences in the mental health of three categories of older adults, namely, those who do not provide grandparental care, those who provide low-intensity care, and those who provide high-intensity care, through linear regression models, a method that captures the mental health of older adults in terms of depression scores; secondly, this paper examines the extent to which different genders affect mental health in terms of whether there is heterogeneity in the group of older people for whom grandparental care has been provided; and finally, this study analyzes the relationship between intergenerational economic support and spiritual comfort and the mental health of the elderly, and examines the mediating role of intergenerational feedback between grandparental care and the mental health of the elderly.

**Figure 1:**
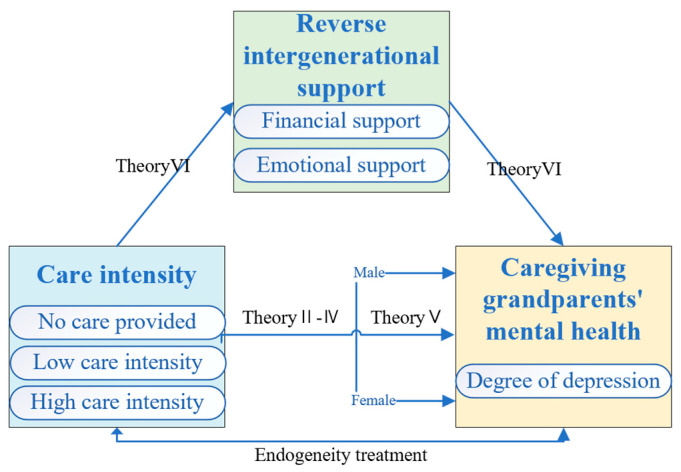
Framework diagram for the study of core variables.

This paper is based on the analytical framework proposed by scholars such as Carlos Cinelli and Chen Lu [46], as shown in Figure 1:

## 3. Materials and Methods

### 3.1. Data Descriptions

**Figure 2:**
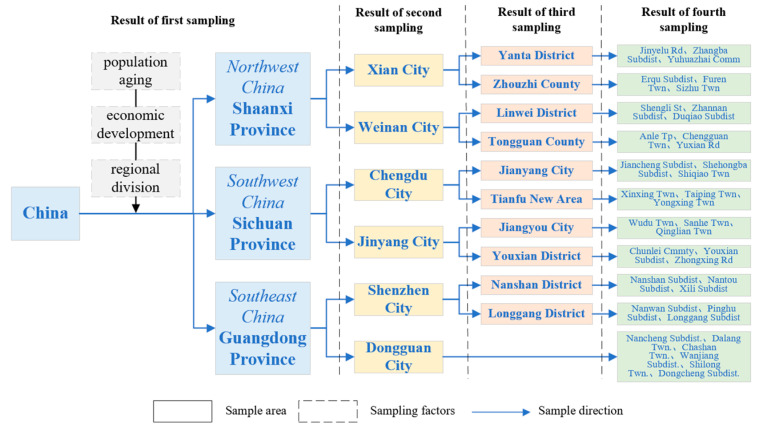
Stratified sampling flowchart.

The data in this paper come from the July–September 2023 Xi’an Jiaotong University Urban and Rural Elderly Family Support and Psychological Condition Research Survey. The survey used a stratified multi-stage random sampling method, and elderly people aged 60 years and above in Shaanxi Province, Sichuan Province, and Guangdong Province, China were selected as the sampling population by considering age, economic, social, and other factors. The survey adopted the Scheaffer sampling formula, and 1600 subjects were selected for the questionnaire survey. According to the principle of stratified sampling, 253 people were selected from Shaanxi Province, 567 people from Sichuan Province, and 780 people from Guangdong Province, and they were taken as the first stratum; additionally, 2 cities were selected as the second stratum by simple random sampling; and 2 counties (districts) were selected as the third stratum by simple random sampling in the selected cities; adopting whole cluster sampling, 3 streets (townships) were selected among counties (districts) as the fourth stratum, as shown in Figure 2; and finally, 1224 valid questionnaires were obtained. At the same time, four representative cases were selected for in-depth interviews to assist in interpreting the research findings. Among them, the questionnaire set up a series of questions specifically for the family support and psychological condition of the elderly, which provided good data support for the research in this paper.

### 3.2. Variable Interpretation

Explained variable. The explanatory variable in this paper is the mental health of older adults, with depression scale scores as a proxy variable. The more authoritative definition of mental health was proposed at the Third International Health Conference in 1946 and refers to the ability to optimize the state of mind of an individual when he or she is not emotionally, physically, and intellectually at odds with others [47]. The mental health performance of the elderly differs from that in other age groups, and physical symptoms such as decreased physical strength and insomnia appear. This paper refers to the research of Yadi Wang [5] (p. 2) and other scholars, utilizing the questionnaire set up to assess the psychological status of “your emotional state last week” in relation to ten questions, including “I am annoyed by some small things”, and so on. The total score of all questions ultimately fell within the assignment range of 10 to 40 points, meaning that the respondents’ mental health was negatively correlated with their depression scale scores.

Explanatory variable. The explanatory variable in this paper is grandparental care. The grandparental care model is a new intergenerational relationship model that distinguishes itself from the traditional model in China and the West, and in her study, Maojuan Che pointed out that grandparents raise the next generation of their children on their behalf, i.e., grandparental care [48]. In order to enhance the explanatory strength, this paper refers to Reinkowski’s research results [49] on the use of the intensity of grandparental caregiving to measure the amount of time and energy that older adults who provide grandparental caregiving invest in that behavior, and the quantification of this dimension will be based on the specific description and frequency of caregiving activities as described by the respondents. This is assessed by asking respondents, “In the past month, how many hours per week on average did you (and your spouse) spend caring for your grandchildren?” If the answer is “0 h/week”, it is assigned a value of 0, indicating no grandparental caregiving; 1 indicates low intensity (greater than 0 less than or equal to 20 h per week); 2 indicates high intensity (greater than 20 h per week).

Control variables. To reduce the omitted variable error, this study draws on the methodology of researchers such as Baoqing Han and Shengjin Wang [28] (p. 4), and selects demographic characteristics and lifestyle variables such as age, gender, household registration, marital status, education level, living arrangements, and number of grandchildren aged 0–6 as the main control variables.

Influence mechanism variables. This study focuses on two key impact mechanism variables: intergenerational financial support and intergenerational emotional comfort. To ensure the quantitative accuracy of intergenerational financial support, the question “How many dollars did your children support you throughout the year?” is asked and responses logarithmically transformed to determine the total amount of financial support from all children. Intergenerational emotional comfort is measured using the question, “On average, how much time per week did your children spend caring for you (and your spouse) in the past month?”, with higher values indicating more frequent emotional comfort. The specific variables are described in Table 1.

### 3.3. Modeling Approach

#### 3.3.1. Baseline Model

This paper constructs a linear regression (OLS) benchmark model, referring to Grossman’s health human capital model [31] (p. 4), taking mental health as the core variable of the study, and adopting grandparental care as the independent variable, to quantitatively analyze the specific impact of grandparental care on the mental health of the elderly. The baseline model is shown in Equation (1):

(1) $$H_{i}=\alpha_{i}+\beta_{i}{GenCare}_{i}+\gamma_{i}X_{i}+\varepsilon_{i}$$

where $H_{i}$ is the mental health of the ith older adult; $\alpha_{i}$ is the intercept term; ${GenCare}_{i}$ is the grandparental caregiving variable for older adults; $X_{i}$ is the control variable; $\beta_{i}$ and $\gamma_{i}$ are the coefficients to be estimated; and $\varepsilon_{i}$ is the random perturbation term.

#### 3.3.2. Propensity Score Matching (PSM)

The grandparental caregiving behaviors of the elderly are usually based on self-selection, and whether or not to implement grandparental caregiving mainly depends on their intergenerational perceptions, economic income status, and occupations, among other factors. Therefore, considering the potential endogeneity of sample selection bias, which makes the regression results lack reference value, this paper adopts the propensity score matching method of “counterfactual estimation” to solve the endogeneity problem caused by self-selection bias. Specifically, we divide the sample into treatment and control groups, and ensure that the two groups have the same range of values for the covariates. For an individual who belongs to the treatment group, similar individuals are found in the control group by matching the covariates and comparing the mental health status of the two groups with or without the provision of grandparental care. In this paper, a Logit model is used to more accurately estimate the probability of an individual being in the treatment group under different conditions, and the model is shown in Equation (2):

(2) $$P\left(X_{i}\right)=\mathrm{Pr}\left[{GenCare}_{i}=1|X_{i}\right]=\frac{exp(\beta_{i})}{1+exp(\beta_{i})}$$

where $P\left(X_{i}\right)$ is the probability that an older person provides grandparental care; $X_{i}$ is a covariate; ${GenCare}_{i}=1$ is the provision of grandparental care; β is a vector of parameters; and $\frac{exp(\beta_{i})}{1+exp(\beta_{i})}$ is a cumulative distribution function. The net effect of the impact of grandparental care on mental health is analyzed by ATT and shown in Equation (3):

(3) $$ATT=E\left[H_{1i}-H_{0i}|{GenCare}_{i}=1\right]=E\left[H_{1i}|{GenCare}_{i}=1\right]-E\left[H_{0i}|{GenCare}_{i}=1\right]$$

where ATT is the average treatment effect; $H_{1i}$ is the mental health status of older adults provided with grandparental care; and $H_{0i}$ is the mental health status of older adults not provided with grandparental care.

## 4. Results

### 4.1. Statistical Description

Among the elderly respondents, 597 elderly people do not provide grandparental care, accounting for 48.77%; 314 elderly people provide low-intensity grandparental care, accounting for 25.65%; and 313 elderly people provide high-intensity grandparental care, accounting for 25.57%. Accordingly, the distribution of each variable is statistically analyzed as shown in Table 2, with the full sample classfied into the following classes: not providing grandparental care, low-intensity grandparental care, and high-intensity grandparental care. For continuous variables, the mean ± standard deviation is statistically analyzed. Ordinal categorical variables are treated as continuous variables. For categorical variables, the proportion (number) of samples with an assigned value of 1 is statistically analyzed.

In terms of type of residence, 51.70% live with their spouse, 28.97% with their children, 18.36% with their grandchildren, and 0.96% with their nanny, as shown in Table 3.

The survey shows that the intensity of care in rural areas is not significantly different from that in urban areas, but the proportion of those not providing care is larger in rural areas, at 27.12% compared to 21.65% in urban areas, as shown in Table 4. Among the respondents who do not provide grandparental care, some grandparents in urban areas participate in day center activities (e.g., chess and dance activities) because of the availability of community services, while in rural areas, they are more likely to choose neighborhood interactions or work in the fields because of the lack of facilities.

### 4.2. Characterization of Changes in the Level of Impact

#### 4.2.1. Base Regression Analysis

In this paper, a stepwise regression method is used to examine the changes in the degree of impact of grandparental care on the mental health of the elderly by sequentially adding care intensity (the reference group is no care provided) and control variables such as age and gender to the model, as shown in Table 5.

The model results show that compared with the elderly who do not provide grandparental care, those who provide low-intensity or high-intensity grandparental care can significantly reduce their level of depression. Moreover, after adding control variables to the model, this conclusion still holds, indicating that providing grandparental care is beneficial to the mental health of the elderly. However, the absolute value of the coefficient for providing high-intensity grandparental care is smaller than that for providing low-intensity grandparental care. This suggests that compared with low-intensity care, high-intensity grandparental care is less conducive to the mental health of the elderly. Research question 1 is thus addressed.

Regarding the control variables, an analysis of Table 5’s (2) reveals that in terms of age, there is a significant positive correlation between age and depression scores, indicating that the mental health of the elderly declines with age. In terms of gender, the depression index of the male elderly is sign ificantly lower than that of the female elderly, suggesting that the mental health of the male elderly is generally better. Additionally, in terms of regional differences, the mental health of older people in urban areas is worse compared to rural areas. In terms of marital status, the mental state of the married elderly is significantly better than that of the divorced, widowed, or other elderly people without a spouse. In terms of education level, there is no significant correlation between educational attainment and depression scores. Taking not living with adult children as the reference, living with children significantly reduces the depression level of the elderly. There is a certain positive correlation between the depression index and the number of grandchildren aged 0–6.

#### 4.2.2. Endogenous Treatment

Research suggests that grandparental caregiving enhances the mental health of older people and that mentally healthy people are more likely to participate in grandparental caregiving. Therefore, the baseline regression model may reveal that there is a correlation between the two, but it does not clarify the causal relationship between them. In this study, in order to eliminate the endogeneity problem caused by bidirectional causality, we adopted the instrumental variable method. According to the literature analysis, the selection of instrumental variables needs to satisfy the following two hypothetical prerequisites: firstly, to ensure that there is a significant correlation between the variable and the core explanatory variables; and secondly, to establish there is no direct influence relationship between the variable and the explanatory variables. On this basis, we rigorously processed and analyzed the relevant data. Based on the appropriate criteria for selecting instrumental variables and referring to the research results of Ku [50] and other related scholars, this paper selects “whether to live with grandchildren” as an instrumental variable. This variable has a high correlation with the phenomenon of reverse parenting among the elderly but has no significant correlation with the mental health of the elderly. Since this article has two dummy variables, namely low care intensity and high care intensity, and only one instrumental variable cannot be directly estimated, we combined two dummy variables into one “Care provided” group for comparison with the “no care provided” group. Therefore, this study utilizes the two-stage least squares (2SLS) method for parameter estimation, and shows the regression results of the first stage, as shown in Table 6.

This study began by analyzing the impact of living with grandchildren on the intensity of caregiving among older adults. The first stage found a significant positive correlation between living with grandchildren and caregiving intensity, indicating that living with grandchildren significantly increases the likelihood of grandparental caregiving among older adults, confirming that the instrumental variables are highly correlated with the endogenous explanatory variables. The second stage of the analysis showed that even after addressing endogeneity, care intensity still had a significant impact on older adults’ mental health. The robust DWH test for model heteroskedasticity, with a *p*-value of 0.0118, could not negate the exogenous relationship between reverse alimony and older adults’ mental health, and identified the presence of endogenous explanatory variables. The weak variable test shows that the value of the model F-statistic is 147.196, which is greater than 10, so there is no weak variable problem.

#### 4.2.3. Selection Bias Treatment—A Study of Propensity Score Matching Estimation

**Figure 3:**
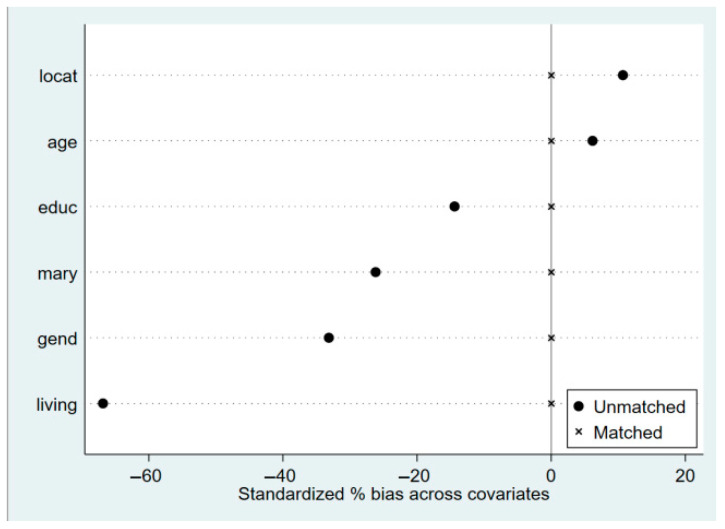
Standardized deviation of variables.

The previous regression results show that grandparental caregiving behaviors enhance the mental health of older adults, but high-intensity grandparental caregiving promotes mental health at a much lower level. However, the process of sample selection for grandparental caregiving is also influenced by a variety of factors. Propensity score matching (PSM) is suitable for observational studies in which randomized controlled trials are not possible and is particularly well suited to dealing with naturally occurring, multifactorial behavioral studies such as grandparental caregiving. It condenses information on multiple covariates into propensity scores, allowing the treatment and control groups to be similar in scores and effectively controlling for confounding factors. Compared with traditional regression analysis, PSM can significantly reduce selection bias, reduce sample heterogeneity through matching, and improve estimation stability and accuracy. In this study, the three methods of neighbor matching, kernel matching, and radius matching were used, and Figure 3 presents the absolute values of bias before and after matching the samples of the impact of grandparental caregiving on mental health. Upon examination, the standardized deviation of most of the variables shrinks to less than 10%, and the sample loss is less than 1%, which strongly guarantees the validity of the estimation results of the impact of grandparental care on the mental health of the elderly. This indicates that the deviations of the observable variables in the treatment and control groups are basically eliminated and the estimation results are valid.

Table 7 demonstrates the results of the ATT treatment using propensity score matching. Notably, all three matching estimates are significant at the 5% statistical level, indicating that the conclusion that grandparental care has a positive effect on the mental health of older adults holds. In fact, after controlling for selective bias and endogeneity issues, the enhancement effect of grandparental caregiving on the mental health impact of older adults is weaker.

### 4.3. Mechanisms of the Impact of Grandparental Caregiving on the Mental Health of Older Persons

From the results analyzed above, we hereby propose two hypotheses about the influencing mechanisms. Based on the intergenerational exchange theory, intergenerational financial support and intergenerational spiritual support may be affected by grandparental caregiving, which in turn affects the mental health of older adults. Accordingly, the following empirical evidence will be used to test the two hypothesized mechanisms.

The data in Table 8 show that controlling for demographic and lifestyle variables, caregiving intensity is positively associated with intergenerational financial support and psychological comfort, which is statistically significant at the 1% level. Lu, J. et al. [51] showed that elderly caregiving for grandchildren enhances reverse reflexivity, increases the chance of socialization, and improves psychological conditions. Table 9 reveals the effects of intergenerational financial support and spiritual solace on the psychological health of the elderly. The findings show that children’s financial support and intergenerational spiritual comfort are negatively correlated with depression scores, suggesting that both significantly reduce depression. The regression results in Table 8 and Table 9 show that grandparental caregiving is in line with the theory of intergenerational exchange as it realizes the enhancement of the mental health of the elderly through the improvement of financial support and spiritual comfort.

### 4.4. Cohort Differences

#### 4.4.1. Subsample Estimation

According to the grandmother hypothesis, elderly women often play a more important role in grandparental care. Therefore, this paper conducts subsample estimations by gender. Step-by-step regression analyses are carried out for both the male and female groups, as shown in Table 10. The results show that the trends and values of the depression index coefficients for women and men vary under different care intensities. Compared with the absence of grandparental care, low-intensity care significantly reduces the depression index for women, having a positive promoting effect on women’s mental health. However, high-intensity care increases the depression index for women, having a negative effect on mental health. In contrast, for men, both low-intensity and high-intensity care reduce the depression index, having a positive promoting effect on men’s mental health. As is well-known, in social roles, women often assume more family responsibilities, such as housework and grandparental care, and such responsibilities largely fall on the shoulders of grandmothers. During this process, grandfathers usually do not assume or only assume an auxiliary role. Therefore, high-intensity grandparental care is instead a burden for women.

#### 4.4.2. Robustness Testing

In this paper, we conduct robustness tests in two ways: (1) Because grandparental caregiving and age are correlated, to correct for the nonlinear effect of age, we add the squared and cubic terms of age to the model. After the inclusion of age squared and cubed, caregiving intensity still has a significant effect on the mental health of older adults (1% significance level), as shown in Table 11, confirming the stability of the baseline regression. (2) The replacement variable method is used to reanalyze the model by including self-assessed psychological status as a proxy indicator. In this process, we use the following question: “How do you rate your psychological status?” To answer the question, given that self-assessed psychological condition is an ordered categorical variable, “very poor, relatively poor, average, relatively good, very good” correspond to scores of 1 to 5, respectively, and the higher the value assigned, the better the older person’s self-assessed psychological condition. In this study, the ordinary least squares (OLS) model and ordered probit model were used for regression analysis of different variables. Table 12 shows that self-assessed psychological status has a significant effect on the mental health of older adults under both model settings, further confirming the robustness of the study findings.

## 5. Discussion

In Chinese society, the role of grandparents is in a transitional stage between “traditional obligations” and “modern autonomy”. On the one hand, intergenerational parenting is still regarded as a “natural choice” by most families, and the caregiving behavior of grandparents has a high functional and emotional value within the family; on the other hand, as the sense of independence rises and retirement life becomes more diversified, support for grandparents’ pursuit of a personal life and their participation in social activities is regarded as a manifestation of “active aging”. Studying its impact on the psychological health of the elderly can not only fill the theoretical gaps in the study of cross-cultural family relations and geriatric psychology but also provide empirical evidence for countries to formulate family support policies and optimize elderly service systems, which is of far-reaching significance in promoting intergenerational harmony and the sustainable development of society. In this paper, we use regression analysis to reveal the complexity of the impact of grandparental caregiving on the mental health of the elderly, adopt the instrumental variable method and propensity to match scores to address the endogeneity problem, and, at the same time, carry out the sub-sample estimation and robustness test. Based on these analyses, we can draw the following conclusions: (1) Grandparental caregiving helps to alleviate depression in the elderly, and positively affects their mental health. After controlling for endogeneity and conducting robustness tests, the findings remain unchanged. This suggests that grandparental caregiving is not only a responsibility but also an emotional support and source of enjoyment. (2) It was found that the positive effects of grandparental caregiving on the mental health of older adults diminish as the intensity of caregiving increases. (3) According to the results of the sub-sample assessment, grandparental caregiving had a positive effect on the mental health of older men and low-intensity caregiving on older women, but high-intensity caregiving had a negative effect on women’s mental health. (4) In the process of grandparental caregiving for the elderly, children indirectly enhance the mental health of the elderly through intergenerational economic support and intergenerational spiritual comfort, emphasizing the mechanism of multiple influences in the intergenerational relationship, which is consistent with the concept of social support, that is, intergenerational support within the family is an important way to enhance the well-being of the elderly. This urges children and grandchildren to strengthen emotional communication with their elders so that their role of emotional support can be maximized and the intensity of grandparental caregiving for older persons can be appropriately reduced.

## 6. Conclusions

Comparing different studies, the results of this study have both similarities and differences in relation to the findings of many other studies. In terms of positive impacts, they are consistent with numerous studies that concluded that grandparental caregiving promotes the mental health of older adults. However, there are differences in the degree of impact and specific mechanisms, with some studies emphasizing that grandparental care improves older adults’ mental health through increased socialization, whereas the present study highlights the role of financial support and emotional comfort more prominently. As a whole, the mental health of older persons is closely related to family and social factors, and this paper makes the following policy recommendations.

(1) At the governmental level, encourage and support the “active aging” of older persons through grandparental care. Government incentives can repair ruptures in family relationships, which makes grandparental support for parents by offspring not only a form of compensation for their parents’ care for them but also an act based on maintaining the bonds of family kinship. In terms of finances, drawing on Japan’s system of “childcare support payments,” and referring to China’s existing models for granting subsidies such as maternity allowances and low-income insurance, and with a mature system of fund management and disbursement in place, the central government allocates a special fund each year, and local governments allocate matching funds in proportion to the number of grandparental caregiving families in their localities, their level of economic development, and so on, to establish a special “subsidy for grandparental caregiving families”. It is technically and administratively less difficult to expand on the existing system of special additional deductions for personal income tax and to make it clear that grandparental caregiving families are eligible for special additional deductions. Implementing stepped subsidies for families with multiple children or female caregivers can effectively reduce the financial burden on grandparental caregiving families, and meets the real needs of China’s aging population and the reduction in the number of children in the country. In the area of social security, further expand the coverage of old-age insurance, refer to the German model of care insurance, include grandparental care in the scope of long-term care insurance payments, increase the number of pilot cities for the long-term care insurance policy, gradually promote it throughout the country, and, in particular, satisfy the diversified needs of older women in the areas of medical care and old-age care. Legislate on old-age security in rural areas [52], organically combining formal social and intergenerational support [53] to ensure that older persons receive adequate material and spiritual support during the process of grandparental care. The pilot work of long-term care insurance has been carried out in some cities and has a certain practical foundation, and the legislation of rural old-age security is an urgent need to solve the problem of rural old-age pension, which is in line with the national strategy of rural revitalization, and it can integrate the resources of all parties, as well as providing legal safeguards for the grandparental care of the elderly in rural areas. At the policy level, the development of a flexible work system that is “maternity-friendly and age-friendly” suggests that employers should provide flexible work options for parents with childcare needs, thereby reducing the intergenerational burden of care for older persons [54]. Flexible work systems have been successfully practiced in foreign countries, and some enterprises in China have also made similar attempts. This system can balance the relationship between family and work, and at the same time help to improve employee satisfaction and loyalty, which has strong feasibility.

(2) At the social level, encourage social organizations, enterprises, and volunteers to provide multi-body “healthy aging” elderly care. First, build a diversified elderly care service supply system with home care as the main focus, and with community as support and institutions as supplements [55]. With the community as the carrier, community workers and volunteers provide special help for the elderly in grandparental care, such as through physical examinations, emotional communication, and assistance in handling, etc., to alleviate the mental pressure on the elderly, and to form a positive interaction between the “care for the elderly” and the “raising of grandchildren”. This will alleviate mental stress among the elderly and truly create a positive interaction between “caring for the elderly” and “raising grandchildren” [56]. China’s communities are relatively well built and have the basic conditions for establishing service centers for the elderly. The problems of staffing and funding can be solved through the purchase of services by the Government and the recruitment of volunteers. Furthermore, strengthen public childcare services and draw on Denmark’s experience in building facilities for the integration of care for the elderly and grandchildren, such as the establishment of specialized places in the community that combine care for the elderly and grandchildren; the construction of facilities for the integration of care for the elderly and grandchildren is in line with the trend of social development, and is capable of improving the efficiency of resource utilization. Encourage younger older persons to provide grandparental care services to other families through “time banks”, where time points can be exchanged for life services or goods of equivalent value, as well as offering social support activities, such as parenting skills instruction and seminars for older persons, to help them to better carry out their daily social activities and to maintain positive attitudes and a high level of personal autonomy [57].

(3) At the family level, to establish the value of “taking care of the elderly” and “raising grandchildren”, and to publicize and popularize the positive significance of grandparental care through the media and the importance of two-way family support. On the one hand, this encourages the elderly to actively take care of their grandchildren. On the other hand, adult children are guided to actively provide financial support, life care, and emotional comfort to their parents [58]. Family old-age care can offer advantages that other old-age care modes cannot match. Family members should pay attention to the emotional needs of the elderly, and provide them with spiritual support by increasing daily communication, community organizations can carry out family interaction activities on a daily basis. Parents and grandparents can, within their means, provide two-way support to enhance family interactions, so that older persons can experience the joys of family life rather than its burdens.

## 7. Limitations

While considering the limitations of the actual survey in this paper, this study still has some limitations. In terms of the research sample, limited by research resources and time, the sample coverage is not broad enough to fully represent the situation of all the elderly in China, especially those in some special areas. In terms of the research methodology, questionnaires and interviews were mainly used, which made it difficult to comprehensively capture the dynamically changing psychological state in the process of grandparental caregiving. In the future, this study will continue to expand the sample to cover older adults from different regions, cultural backgrounds, and family structures; at the same time, it will adopt professional mental health monitoring techniques, such as the Hamilton Depression Scale (HAMD), to more scientifically and objectively measure the psychological status of older adults, and it will strengthen the dynamic tracking research on the process of grandparental caregiving by individuals, in order to analyze the mechanisms that affect the psychological health of older adults.

## Figures and Tables

**Table 1:** Variable settings and descriptions.

Variable Category	Variable Name	Description of Variables
Explained variable	Depression score	Continuous variables. According to the 10 questions on the scale, the options “rarely or not at all (<1 day)”, “not too much (1–2 days)”, “sometimes or half the time (3–4 days)”, “most of the time (5–7 days)” were assigned scores of 1, 2, 3, and 4 respectively; for the positive questions, “I am hopeful for the future” and “I am happy”, the scores were reversed.
Explanatory variable	Care intensity	Dummy variables. “How many hours per week, on average, did you (and your spouse) spend caring for your grandchildren in the past 1 month?”, 0 = 0 h/week, 1 = 0 to 20 h (low care intensity), 2 = more than 20 h (high care intensity).
Control variables	Age	Continuous variable.
	Gender	Dummy variable, 0 = female, 1 = male.
	Household registration	Dummy variable, 0 = rural, 1 = urban.
	Marital status	Dummy variables, 0 = unmarried, including those who have never married, divorced, widowed, etc., 1 = in marriage.
	Educational level	Ordinal categorical variables, 1 = elementary school and below, 2 = junior high school, 3 = high school/middle school, 4 = junior college, 5 = bachelor’s degree and above.
	Living arrangements	Dummy variable. “Whether grandparents live with their adult children?”, 0 = false, 1 = true.
	Number of grandchildren aged 0–6	Continuous variable.
Influence mechanism variables	Intergenerational economic support	Continuous variable based on the question, “How many dollars did your children support you throughout the year?” This question was log-transformed to total financial support from all children.
	Intergenerational emotional comfort	Continuous variable based on the question, “On average, how much time per week did your children spend caring for you (and your spouse) in the past month?” The question is taken to mean the actual amount.

**Table 2:** Descriptive statistics of relevant variables.

Variable		Full Sample (Number = 1224)	Not Providing Grandparental Care (Number = 597)	Low Care Intensity (Number = 314)	High Care Intensity (Number = 313)
Continuous variables	Depression score	17.63 ± 5.53	18.28 ± 5.54	16.44 ± 5.46	17.58 ± 5.38
	Age	70.10 ± 6.92	70.94 ± 7.30	69.94 ± 6.95	68.64 ± 5.82
	Financial support from children (in CNY 1000)	5.91 ± 11.00	5.09 ± 9.92	7.56 ± 13.33	5.82 ± 10.21
	How many hours do your children spend on average per week providing care for you?	14.89 ± 30.65	12.24 ± 33.16	11.38 ± 16.86	23.47 ± 34.55
	How many hours do you spend on average per week providing care for your grandchildren?	18.43 ± 42.37	0.00 ± 0.00	9.98 ± 6.09	62.06 ± 66.08
	Self-evaluated mental status	3.90 ± 0.88	3.83 ± 0.87	3.96 ± 0.84	3.95 ± 0.94
	Self-evaluated physical status	3.60 ± 0.97	3.51 ± 0.94	3.71 ± 0.95	3.65 ± 1.04
	Number of grandchildren aged 0–6	0.32 ± 0.79	0.13 ± 0.45	0.47 ± 1.08	0.51 ± 0.88
Ordinal categorical variables	Education level	2.09 ± 1.17	2.03 ± 1.21	2.14 ± 1.11	2.17 ± 1.13
Dummy variables	Gender	50.49% (618)	54.27% (324)	51.59% (162)	42.17% (132)
	Household registration	46.5% (569)	44.39% (265)	47.77% (150)	48.88% (153)
	Marital status	75.41% (923)	71.36% (426)	79.30% (249)	79.23% (248)
	Living arrangements	39.46% (483)	31.49% (188)	39.81% (125)	54.31% (170)

**Table 3:** Residential type of the elderly.

Option	Frequency (Person)	Percentage (%)
Live with Spouse	862	51.70
Live with Children	483	28.97
Live with Grandchildren	306	18.36
Live with Nanny	16	0.96

**Table 4:** Comparison of care intensity between urban and rural areas.

Care Intensity (h/Week)	Urban	Rural	Total
No Care Provided	21.65%	27.12%	48.77%
Greater than 0 less than or equal to 20	12.42%	13.24%	25.66%
More than 20	12.59%	12.58%	25.17%
Total	46.66%	52.94%	100.00%

**Table 5:** Stepwise regression analysis results.

Variable		Depression Score (1)	Depression Score (2)
Care Intensity (Reference group: no care provided)	Low Care Intensity	−0.78 *** (0.034)	−0.73 *** (0.035)
	High Care Intensity	−0.16 *** (0.042)	−0.15 *** (0.041)
Age			0.05 *** (0.018)
Gender (Reference group: male)			0.12 *** (0.032)
Household Registration (Reference group: rural)			0.13 *** (0.039)
Marital Status (Reference group: unmarried)			−1.34 *** (0.038)
Education Level			−0.14 (0.092)
Living Arrangements (Reference group: not living with parents)			−0.32 ** (0.140)
Number of Grandchildren Aged 0–6			0.13 * (0.075)
_cons		18.28 *** (0.087)	16.03 *** (0.182)
N		1224	1223
R-squared		0.0042	0.0215

Note: Robust standard errors in parentheses, * *p* < 0.1, ** *p* < 0.05, *** *p* < 0.01.

**Table 6:** Endogeneity treatment—instrumental variable method (2SLS).

Variable	First Stage	Second Stage
	Care Provided (Reference Group: No Care Provided)	Depression Score
Whether to live with grandchildren (Reference group: false)	0.38 *** (0.03)	
Care provided (Reference group: no care provided)		−1.78 ** (0.76)
Other variables	Controlled	Controlled
N	1084	1084
The first-stage F-value	26.84	
DWH Test	F = 2.44308 (*p* = 0.0118)	
Weak instrument test	F = 147.196 (*p* = 0.0000)	

Note: Robust standard errors in parentheses, ** *p* < 0.05, *** *p* < 0.01.

**Table 7:** ATT treatment results are based on propensity score matching.

Variable	Observed Coefficient	Robust Standard Error	T-Value	*p*-Value	95% Confidence Interval
Depression Score					
Nearest Neighbor Matching *	−0.3366961	0.2452017	−1.37	0.017	−0.1438903–0.8172826
Kernel Matching	−0.117998	0.2341148	−0.50	0.001	−0.3408585–0.5768546
Radius Matching	−0.117998	0.2341148	−0.50	0.000	−0.3408585–0.5768546

* Nearest neighbor matching uses one-to-one matching; radius matching employs a caliper of 0.01. Kernel matching uses the default bandwidth, with replications = 500.

**Table 8:** Effects of care intensity on intergenerational economic support and intergenerational emotional comfort.

Variable		Children’s Financial Support (Logarithm Function with +1)	Average Weekly Care Time by Children for You
Care Intensity (Reference group: no care provided)	Low Care Intensity	0.237 *** (0.092)	5.411 *** (2.097)
	High Care Intensity	0.250 *** (0.097)	10.691 *** (2.163)
Other Variables		Controlled	Controlled
_cons		7.830 *** (0.46)	49.099 *** (9.98)
N		649	1220
R-squared		0.0835	0.0957

Note: Robust standard errors in parentheses, *** *p* < 0.01.

**Table 9:** Effects of intergenerational support on elderly mental health.

Variable	Depression Score	
Children’s Financial Support (Log)	−0.02 *** (0.006)	
Weekly Care Time by Children		−0.39 *** (0.119)
Other Variables	Controlled	Controlled
_cons	1.56 *** (0.19)	7.77 *** (3.03)
N	1224	1224
R-squared	0.0056	0.0049

Note: Robust standard errors in parentheses, *** *p* < 0.01.

**Table 10:** Subsample estimates of the impact of grandparental care on the mental health of elderly adults.

Variable		Female		Male	
		Depression Score (1)	Depression Score (2)	Depression Score (1)	Depression Score (2)
Care Intensity (Reference group: no care provided)	Low Care Intensity	−0.80 *** (0.074)	−0.54 *** (0.076)	−0.87 *** (0.079)	−0.67 *** (0.077)
	High Care Intensity	0.14 *** (0.044)	0.13 *** (0.046)	−0.34 *** (0.041)	−0.30 *** (0.048)
Other Variables			Controlled		Controlled
_cons		18.06 *** (0.117)	19.54 *** (0.221)	18.54 *** (0.191)	13.89 *** (0.245)
N		618	618	606	606
R-squared		0.0024	0.0259	0.0033	0.0311

Note: Robust standard errors in parentheses, *** *p* < 0.01.

**Table 11:** Robustness test with nonlinear age variables.

Variable		Depression Score (1)	Depression Score (2)	Depression Score (3)
Care Intensity (Reference group: no care provided)	Low Care Intensity	−1.02 *** (0.393)	−1.02 *** (0.394)	−1.02 *** (0.395)
	High Care Intensity	−1.03 *** (0.399)	−1.03 *** (0.399)	−1.03 *** (0.399)
Age		0.061 *** (0.023)	0.952 *** (0.354)	9.474 ** (4.190)
Age Squared/100 *			−0.006 *** (0.002)	−0.123 ** (0.057)
Age Cubed/1000				0.001 ** (0.000)
Controlled Variables		Controlled	Controlled	Controlled
_cons		17.035 *** (1.800)	−33.2 *** (12.832)	−221.42 ** (101.24)
N		1223	1223	1223
R-squared		0.0251	0.0297	0.0328

* To reduce estimation errors, the age-squared and cubed terms are divided by 100 and 1000, respectively. Robust standard errors in parentheses, ** *p* < 0.05, *** *p* < 0.01.

**Table 12:** Robustness test with alternative dependent variable.

Variable	Care Intensity (Reference Group: No Care Provided)		Controlled Variables	_cons	N	R-Squared
	Low Care Intensity	High Care Intensity				
Self-rated Mental Health	0.156 *** (0.060)	0.170 *** (0.066)	Controlled	3.954 *** (0.293)	1223	0.0534

Note: Cut1–Cut4 regression results from the ordered probit model are omitted. Robust standard errors in parentheses, *** *p* < 0.01.

## Data Availability

The data presented in this study are available on request from the corresponding author due to the data is derived from a questionnaire survey and the policies and confidentiality agreements followed by the previous survey project, it involves a lot of private information of the respondents.

## Abbreviations

The following abbreviations are used in this manuscript:

PSM	Propensity Score Matching
OLS	Ordinary Least Squares
ATT	Average Treatment Effects on Treated
2SLS	Two-Stage Least Square
